# Oocyte-Specific Deletion of *Slc6a9* Encoding the GLYT1 Glycine Transporter Eliminates Glycine Transport in Mouse Preimplantation Embryos and Their Ability to Counter Hypertonic Stress

**DOI:** 10.3390/cells12202500

**Published:** 2023-10-21

**Authors:** Allison K. Tscherner, Taylor McClatchie, Gracia Kaboba, Detlev Boison, Jay M. Baltz

**Affiliations:** 1 Ottawa Hospital Research Institute, Ottawa, ON K1H 8L6, Canadatmcclatchie@ohri.ca (T.M.); graciakaboba12@gmail.com (G.K.); 2 Department of Obstetrics and Gynecology, Faculty of Medicine, University of Ottawa, Ottawa, ON K1Y 8L6, Canada; 3 Department of Neurosurgery, Robert Wood Johnson Medical School, Rutgers University, Piscataway, NJ 08854, USA; db1114@rwjms.rutgers.edu; 4 Department of Cellular and Molecular Medicine, Faculty of Medicine, University of Ottawa, Ottawa, ON K1Y 8L6, Canada

**Keywords:** oocyte, preimplantation embryo, glycine, transport, cell volume, knockout, GLYT1, mouse, cumulus, osmolyte

## Abstract

Early preimplantation mouse embryos are sensitive to increased osmolarity, which can block their development. To overcome this, they accumulate organic osmolytes to maintain cell volume. The main organic osmolyte used by early mouse embryos is glycine. Glycine is transported during the mature egg and 1-cell to 4-cell embryo stages by a transporter identified as GLYT1, encoded by the *Slc6a9* gene. Here, we have produced an oocyte-specific knockout of *Slc6a9* by crossing mice that have a segment of the gene flanked by LoxP elements with transgenic mice expressing iCre driven by the oocyte-specific *Gdf*9 promoter. *Slc6a9* null oocytes failed to develop glycine transport activity during meiotic maturation. However, females with these oocytes were fertile. When enclosed in their cumulus-oocyte complex, *Slc6a9* null oocytes could accumulate glycine via GLYT1 transport in their coupled cumulus cells, which may support female fertility in vivo. In vitro, embryos derived from *Slc6a9* null oocytes displayed a clear phenotype. While glycine rescued complete preimplantation development of wild type embryos from increased osmolarity, embryos derived from null oocytes failed to develop past the 2-cell stage even with glycine. Thus, *Slc6a9* is required for glycine transport and protection against increased osmolarity in mouse eggs and early embryos.

## 1. Introduction

Early preimplantation mouse embryos are particularly susceptible to relatively small increases in external osmolarity [1,2,3]. Increases in osmolarity can result in complete blocks to development in vitro, including the classic “2-cell block” in mouse embryos [2,4]. Because mammalian cells regulate their volumes by adjusting intracellular osmotic pressure [5], such sensitivity to hypertonicity was linked to disruption of their ability to maintain cell volume within an acceptable range [1,6,7].

The acute response of mammalian cells to a decrease in cell volume is to activate a set of transporters that act to accumulate inorganic ions, mainly Na^+^, Cl^−^ and K^+^ [5]. Such mechanisms are active in early mouse preimplantation embryos, principally functionally coupled sodium-hydrogen and bicarbonate-chloride exchangers [1,8,9,10,11]. However, accumulation of sufficient inorganic ions to balance external osmotic pressure can be damaging in the long term, which has led many somatic cells to employ mechanisms to accumulate benign “organic osmolytes” that can replace a portion of the inorganic ions while providing osmotic support [12]. Characteristic organic osmolyte transporters operate in somatic cells [13], but early embryos instead have unique organic osmolyte transporters, with the main one relying on glycine transport [1,3,7,14].

The glycine transport that is present in early embryos was attributed to “System Gly,” a classic transport activity characterized as accepting glycine and sarcosine (N-methylglycine) as high-affinity substrates and dependence on cotransport of Na^+^ and Cl^−^ with the organic substrate [15,16]. System Gly activity was found to be mediated at the molecular level by the product of the *Slc6a9* gene (initially named *Glyt1*) with the System Gly transporter then designated GLYT1 [17]. Early mouse embryos have robust glycine transport that is competitively inhibited by sarcosine and is dependent on the Na^+^ and Cl^−^ gradients [15,16], consistent with GLYT1 activity.

GLYT1 activity first appears during meiotic maturation of the oocyte. Germinal vesicle stage oocytes transport little or no glycine, but glycine transport reaches its maximum rate within several hours of ovulation being triggered in vivo or the oocyte being removed from the follicle in vitro [18,19,20]. After GLYT1 is activated, intracellular soluble glycine levels increase from undetectable in the GV oocyte to very high levels of ~20–30 mM in second meiotic metaphase (MII) eggs and early preimplantation embryos, which is sufficient to provide substantial osmotic support [18,21]. The apparent GLYT1 activity is present up to about the 4-cell stage, after which it again becomes undetectable. The high intracellular glycine levels also remain through the 2-cell stage but disappear by the morula stage [18]. Thus, GLYT1-mediated glycine transport appears to be present during the stages when embryos are most susceptible to hypertonic stress and support the accumulation of large amounts of intracellular glycine to act as an organic osmolyte. Glycine transport is present in early human embryos where it may perform a similar function [22].

Glycine rescues the development of 1-cell mouse embryos to blastocysts in hypertonic media [7,14]. This ability has been attributed to GLYT1 because virtually all detectable glycine transport from the mature egg through the 4-cell stage has transport characteristics consistent with GLYT1 (above) and because the ability of glycine to rescue development at increased osmolarity was eliminated in the presence of a small molecule inhibitor that was developed to be highly selective against GLYT1 [23]. However, it has not been shown directly that *Slc6a9* expression is necessary for glycine transport in early mouse embryos nor whether it is required for glycine to have a protective effect on embryo development under hypertonic conditions. Furthermore, whether lack of *Slc6a9* or GLYT1 activity in oocytes affects female fertility is not known. Here, we have created a conditional knockout of *Slc6a9* in oocytes to resolve these questions.

## 2. Materials and Methods

### 2.1. Chemicals and Media

Chemicals and reagents were obtained from Sigma-Aldrich (Oakville, ON, Canada) unless otherwise specified. In vitro maturation of oocytes was done in Minimal Essential Medium Alpha (MEMα; catalog #12561–056, Life Technologies, Burlington, ON, Canada) that was supplemented with cold water-soluble polyvinyl alcohol (PVA). Preimplantation embryos were cultured in modified potassium-supplemented Simplex Optimized Medium (mKSOM) with PVA instead of bovine serum albumin and omitting glutamine [24]. The mKSOM medium contains NaCl (95 mM), KCl (2.5 mM), KH_2_PO_4_ (0.35 mM), MgSO_4_·7H_2_O (0.2 mM), Na lactate (10 mM), Glucose (0.2 mM), Na pyruvate (0.2 mM), NaHCO_3_ (25 mM), CaCl_2_.2H_2_O) (1.7 mM), tetrasodium ethylenediaminetetraacetic acid (EDTA) (0.01 mM), K penicillin G (0.16 mM), Streptomycin SO_4_ (0.03 mM), and 1mg/mL PVA. mKSOM was used at 37 °C in 5% CO_2_ in air at 100% humidity. Collection of oocytes and 1-cell embryos was done using Hepes-mKSOM that was identical to mKSOM except 21 mM 4-(2-hydroxyethyl)-1-piperazineethanesulfonic acid (Hepes) was added and NaHCO_3_ was reduced to 4 mM, and pH was adjusted to 7.4 at room temperature using NaOH. Components of media were embryo-tested or cell culture-tested grade. The osmolarities were 250 mOsM for mKSOM and 240 mOsM for Hepes-mKSOM (±5 mOsM). Where hypertonic mKSOM was used, its osmolarity was increased using the inert trisaccharide D(+)-raffinose as previously described [14].

### 2.2. Nomenclature

Genes are identified using their standard nomenclature. Mouse lines are identified by the standard nomenclature used in the literature. The shorthand designations of each mouse line and their derivatives are used throughout (see below). For most lines, the gene and mouse line designations correspond, including Gdf9-Cre, Amhr2-Cre, and Cyp19-Cre. However, where LoxP sites were inserted in the *Slc6a9* gene, the designations use “Glyt1,” which was the original designation of this gene and still commonly used in the literature. To be consistent with previously published work [25,26], we have used the Glyt1 nomenclature for this line and its derivatives, while referring to the gene itself as *Slc6a9*.

### 2.3. Animals

The University of Ottawa Animal Care Committee approved the animal usage and breeding protocols (approval numbers 3347 for breeding and 3348 for experiments), which comply with the regulations of the Canadian Council on Animal Care. Mice were maintained on a 12 h light:dark cycle with unrestricted access to water and Teklad Global 18% protein rodent diet 2018 (Envigo, Indianapolis, IN, USA).

B6D2F1 (BDF1) male mice were obtained from Charles River Canada (St. Constant, QC, Canada) and used for mating with females to produce offspring for fertility testing and 1-cell stage embryos for culture.

Glyt1tm1.2fl/fl mice (shorthand: Glyt1^fl/fl^) have LoxP sites that flank *Slc6a9* gene exons 5–11 and were produced as previously described [25,26]. They were maintained on the C57Bl6 background.

Tg(Gdf9-icre)5092Coo/J (shorthand: Gdf9-iCre) mice express iCre driven by the mouse *Gdf9* gene promoter, restricting iCre exclusively to the oocyte [27]. They were a gift from Dr. Barbara Vanderhyden (Ottawa, ON, Canada) and were maintained on the C57Bl6 background.

Amhr2tm3(cre)Bhr (shorthand: Amhr2-Cre) mice express Cre driven by the *Amhr2* promoter [28]. *Amhr2* is highly expressed in the granulosa cells of preantral and antral follicles as well as other reproductive tissues of Müllerian duct origin [29,30]. They were a gift from Dr. Barbara Vanderhyden (Ottawa, ON) and were maintained on the C57Bl6 background.

Tg(CYP19A1-cre)1Jri (shorthand: Cyp19-Cre) mice express iCre driven by the *Cyp19a1* promoter. *Cyp19a1* is highly expressed in granulosa cells of preantral and antral follicles [31,32]. Cyp19-Cre mice that also had the *Smarca4 (*a.k.a. *Brg1*) gene flanked by LoxP sites were a gift from Dr. Barbara Vanderhyden (Ottawa, ON, Canada). These were first crossed with C57Bl6/J mice to eliminate *Smarca4*^fl/fl^ and then were maintained on the C57Bl6 background.

### 2.4. Production of Conditional Knockouts

The Gdf9-iCre:Glyt1^fl/fl^ line was produced by crossing Gdf9-iCre with Glyt1^fl/fl^ mice. Because Gdf9-iCre is expressed in oocytes, this transgene should normally be transmitted only through males to prevent heterozygous loss of Glyt1 that occurs in offspring of GDF9-iCre:Glyt1^fl/fl^ females mated with wild type males [33]. However, in the initial cycles of breeding, the Gdf9-iCre transgene was also transmitted through females to more rapidly increase the breeding stock. In this case, only Glyt1^fl/fl^ offspring were retained and those that were Glyt1^fl/−^ were not bred further (except for producing Amhr2-Cre:Glyt1^fl/−^ mice; see below). Generally, Gdf9-iCre:Glyt1^fl/+^ males from the initial crosses were mated to Glyt1^fl/fl^ females to provide Gdf9-iCre:Glyt1^fl/fl^ males, which were paired with Glyt1^fl/fl^ females and maintained as breeding stock. Since the males carry one copy of the iCre transgene, the offspring were either Gdf9-iCre:Glyt1^fl/fl^ or Glyt1^fl/fl^. Females of these genotypes were used as the experimental animals (Glyt1 knockout oocytes) and controls (Glyt1 wild type oocytes), respectively.

The Amhr2-Cre:Glyt1^fl/fl^ line was produced by crossing Amhr2-Cre with Glyt1^fl/fl^ mice to produce Amhr2-Cre:Glyt1^fl/+^ offspring. These were mated to Glyt1^fl/fl^ to produce Amhr2-Cre:Glyt1^fl/fl^ offspring, which were then paired with Glyt1^fl/fl^ and maintained as breeding pairs. Because there was no oocyte expression of Cre, both males and females of the Amhr2-Cre:Glyt1^fl/fl^ genotype could be used for breeding to produce offspring that were either Amhr2-Cre:Glyt1^fl/fl^ or Glyt1^fl/fl^. Females of these genotypes were used as the experimental animals (Cre positive) and controls (Cre negative), respectively.

The Cyp19-Cre:Glyt1^fl/fl^ line was produced using the same breeding scheme as the Amhr2-Cre:Glyt1^fl/fl^ line. Offspring of the breeding pairs were either Cyp19-Cre:Glyt1^fl/fl^ (experimental) or Glyt^fl/fl^ (control).

Amhr2-Cre:Glyt1^fl/−^ mice were produced by crossing Glyt1^+/−^ mice with Amhr2-Cre:Glyt1^fl/fl^ mice. The Glyt1^+/−^ mice were produced from mating Gdf9-iCre:Glyt1^fl/fl^ females with BDF1 males. Since Gdf9-iCre:Glyt1^fl/fl^ females have both copies of Glyt1 inactivated in their oocytes, they produce offspring with one copy of Glyt1 inactivated (Glyt1^+/−^) when mated with wild type males. Those Glyt1^+/−^ offspring that did not inherit Gdf9-Cre were selected and mated with Amhr2-Cre:Glyt1^fl/fl^ mice to produce offspring with Amhr2-Cre:Glyt1^fl/+^ and Amhr2-Cre:Glyt1^fl/−^ genotypes as well as the corresponding Amhr2-Cre negative genotypes (Glyt1^fl/+^ and Glyt1^fl/−^).

### 2.5. Genotyping

Genotyping was done using ear notch samples. PCR products were visualized on 1.4% agarose gels with ethidium bromide. Band sizes were estimated from DNA ladders (ThermoFisher Scientific #SM0403). The lower set of resolved marker bands range from 100 bp to 900 bp at 100 bp intervals and then 1031 bp; the upper set was 1500 bp, 2000 bp, and 2500 bp, then 3000 bp to 10,000 bp at 1000 bp intervals, the upper range of which was generally not fully resolved. Glyt1^fl/fl^ genotyping of offspring was done using the 17854 sense primer (5′-TGG CAC CTC TCT GAG TGT GC-3′) and 17672 antisense primer (5′- TTC CAG GAC ATC CAG ATG ATG C - 3′) that yield bands of 258 bp for Glyt1^fl/fl^ and 182 bp for wild type.

Determinations of whether Cre-mediated deletion of a segment of *Slc6a9* had occurred in Glyt1^fl/fl^ tissues was done using the o250 sense primer (5′-CCC ATG CCC AGA TCC ATG C-3′) targeted 5′ to the LoxP site and the o228 antisense primer targeting *Slc6a9* sequence 3′ to the PGK-neomycin cassette (5′-GTC AAC CTG ACT CCT AGC CCT GTA CC-3′) as previously described [25]. In the absence of Cre, this yields a product of ~6600 bp. With successful Cre-mediated deletion, a band of ~450 bp is produced instead. This was performed on ovary and liver samples to assess the effects of Cre targeted to granulosa cells.

The Gdf9-iCre transgene was detected using the primer sets specified by The Jackson Laboratory (https://www.jax.org/Protocol?stockNumber=011062&protocolID=17814 (accessed on 4 September 2023)). The iCre transgene was detected with forward primer 25494 (5′-GGC ATG CTT GAG GTC TGA TTA C-3′) and reverse primer 21218 (5′-CAG GTT TTG GTG CAC AGT CA-3′), which were multiplexed with the Internal Positive Control (targeting the mouse *Il2* gene) forward primer oIMR7338 (5′-CTA GGC CAC AGA ATT GAA AGA TCT-3′) and reverse primer oIMR7339 (5′-GTA GGT GGA AAT TCT AGC ATC ATC C-3′). The Cre transgene primers yield a 200 bp product and the positive control primers yield 324 bp.

The Amhr2-Cre transgene was detected with the forward primer AMHR2Cre FWD (5′-CTC TGG TGT AGC TGA TGA TC-3′) and reverse primer AMHR2Cre REV (5′-TAA TCG CCA TCT TCC AGC AG -3′), which were multiplexed with the Internal Positive Control primer IMR0015 (5′-CAA ATG TTG CTT GTC TGG TG-3′) and reverse primer IMR0016 (5′-GTC AGT CGA GTG CAC AGT TT-3′). The Cre transgene was indicated by a 340 bp product and the positive control product was 206 bp.

The Cyp19-Cre transgene was detected with the forward primer Cyp19CreF (5′-ACTTGGTCAAAGTCAGTGCG-3′) and reverse primer Cyp19CreR (5′-CCTGGTGCAAGCTGAACAAC-3′), which yields a 290 bp product. No internal control was used for Cyp19-Cre, so these primers were always paired with the Glyt1^fl/fl^ primers (above) to ensure DNA quality.

### 2.6. GV Oocytes and 1-Cell Stage Embryos

To obtain germinal vesicle stage (GV) oocytes, females were superovulated with 5 IU equine chorionic gonadotropin (eCG, Prospec, Sturgeon County, AB, Canada) administered by intraperitoneal (IP) injection. Ovaries were excised and minced in Hepes-KSOM at 44–46 h post-eCG. Cumulus-oocyte complexes (COCs) were collected, and cumulus cells were removed by repeated pipetting to obtain denuded oocytes.

One-cell stage embryos were obtained by inducing ovulation with an IP injection of 5 IU human chorionic gonadotropin (hCG, Prospec) at 47 h post-eCG. Females were then caged overnight with BDF1 males (Charles River Canada) following hCG injection. Embryos were obtained 21–24 h post-hCG by flushing oviducts with Hepes-KSOM medium using a blunt-end syringe.

### 2.7. Glycine Transport Measurements

The methods for measuring GLYT1 activity have been extensively described and validated previously [18,19,20,23,34]. [^3^H]glycine ([2-^3^H]glycine; 40–60 Ci/mmole) was obtained from Perkin-Elmer (Waltham, MA) and used where indicated. During the course of this work, [^3^H]glycine that passed quality control tests (see below) became unavailable from this source. After that, [^3^H]glycine ([2-^3^H]glycine; 2.4 Ci/mmole) was obtained from Movarek (Brea, CA) and used where indicated. Each new stock of [^3^H]-glycine was subject to a quality control test that confirmed that transport of 1 µM [^3^H]-glycine into mouse 1-cell embryos was inhibited by >90% in the presence of 5 mM unlabelled glycine.

Glycine transport into individual denuded oocytes was measured by incubating oocytes in 3 µM [^3^H]glycine (Perkin-Elmer) after they had been in vitro matured overnight in MEMα medium to MII eggs to allow GLYT1 activity to reach maximum levels. Glycine transport into COCs was measured using 1 µM [^3^H]glycine from Perkin-Elmer or 10 µM of the lower specific activity [^3^H]glycine from Movarek. It was confirmed using remaining Perkin-Elmer stock that had passed the quality control test that essentially the same results were obtained when using [^3^H]glycine from either source. Glycine transport measurements were carried out in 50 µL drops of mKSOM under mineral oil with a 10 min incubation with [^3^H]glycine.

After incubation with [^3^H]glycine, denuded oocytes or COCs were washed 7× in ice-cold Hepes-mKSOM and placed into scintillation vials with 4 mL Scintiverse BD scintillation fluid (Fisher Scientific, Pittsburgh, PA). An LS6500 liquid scintillation counter (Beckman Coulter, Brea, CA) set at a 5 min counting period was used to quantify the accumulated [^3^H]glycine. CPM were converted to molar amounts of [^3^H]glycine using standard curves constructed from serial dilutions of [^3^H]glycine stocks. Backgrounds with no added [^3^H]glycine were subtracted from each reading. Rates of glycine transport were reported as fmole [^3^H]-glycine per oocyte per min per µM [^3^H]-glycine in the incubation medium.

Transfer of [^3^H]glycine from cumulus cells into the enclosed oocyte was assessed by incubating both COCs and denuded GV oocytes for 4 h in mKSOM with 10 µM [^3^H]glycine (Movarek). After the incubation, cumulus cells were removed from the oocyte in COCs by repeated pipetting though a narrow bore pipette. The amount of [^3^H]glycine that had been accumulated in oocytes that were within COCs vs. those that were denuded oocytes during the 4 h incubation was then determined by liquid scintillation counting as described above.

### 2.8. Data Analysis

Data were analyzed and graphed using GraphPad Prism 9 or 10 (San Diego, CA, USA). The statistical significance of differences between means was determined by one-way ANOVA with Tukey’s multiple comparisons test for more than two groups. A *t*-test was used for two groups (or a Mann-Whitney test for non-normally distributed data). One-sample *t*-tests were used to test for significant difference of means from zero. Statistical significance was taken to be *p* < 0.05.

## 3. Results

### 3.1. Oocyte-Specific Glyt1 Deletion

We first confirmed that Gdf9-iCre:Glyt1^fl/fl^ females produced Glyt1 null oocytes. To avoid genotyping individual oocytes, Gdf9-iCre:Glyt1^fl/fl^ females were mated with wild type (BDF1) proven fertile males and their offspring were genotyped. If all oocytes in Gdf9-iCre:Glyt1^fl/fl^ females had a disrupted Glyt1 (*Slc6a9*) gene, then all offspring from this mating would be heterozygous and have an *Slc6a9*^+/−^ genotype. Four Gdf9-iCre:Glyt1^fl/fl^ female x BDF1 male matings produced litters of 3, 6, 9, and 7 pups, all 25 of which had an *Slc6a9*^+/−^ genotype. Three control Glyt1^fl/fl^ females were mated with BDF1 males and produced litters of 6, 8, and 9 pups, all 23 of which had an *Slc6a9*^+/+^ genotype. Therefore, the genotypes of the offspring indicate that Gdf9-iCre:Glyt1^fl/fl^ females only produce oocytes in which both copies of Glyt1 have been disrupted.

**Figure 1:**
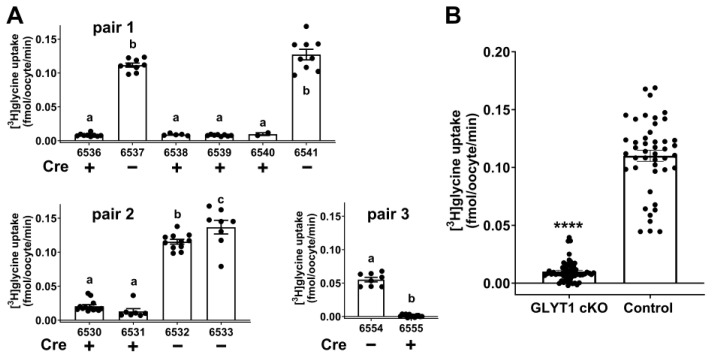
Glycine transport into individual denuded oocytes from females of either Gdf9-iCre:Glyt1^fl/fl^ or Glyt1^fl/fl^ genotypes. (**A**) Gdf9-iCre:Glyt1^fl/fl^ males were mated with Glyt1^fl/fl^ females. Oocytes were collected from the female offspring of three such pairs (pair 1, pair2, pair 3) and the transport of [^3^H]glycine measured for each oocyte after in vitro maturation to MII eggs. The genotypes of the offspring were either Glyt1^fl/fl^ (6537, 6541, 6532, 6533, 6554) or Gdf9-iCre:Glyt1^fl/fl^ (6536, 6538, 6539, 6540, 6530, 6531, 6555). All oocytes from the Cre positive females exhibited very low glycine transport, while those from Cre negative females exhibited high levels of glycine transport. The rates of glycine transport differed significantly between oocytes from Cre positive vs. Cre negative females (*p* < 0.0001 for a vs. b or c within each panel by ANOVA with Tukey’s multiple comparisons test for pairs 1 and 2, or unpaired *t*-test for pair 3; for pair 2, uptake by the oocytes from the two Cre negative females also differed significantly, b vs. c, *p* = 0.026). (**B**) Data for all individual oocytes shown in (**A**) were pooled. Overall, there was a highly significant difference between the Cre positive (Glyt1 cKO) and Cre negative (control) oocytes (*p* < 0.0001, Mann-Whitney test). Bars indicate the mean ± s.e.m.

We next assessed glycine transport in individual denuded oocytes. Glycine transport into individual oocytes obtained from Gdf9-iCre:Glyt1^fl/fl^ or from control Glyt1^fl/fl^ females was measured using [^3^H]glycine. Breeding pairs of Gdf9-iCre:Glyt1^fl/fl^ males mated with Glyt1^fl/fl^ females produced litters with both Glyt1^fl/fl^ (Cre negative control) and Gdf9-iCre:Glyt1^fl/fl^ (Cre positive) offspring. Three different breeding pairs produced the litters used in these experiments. All females in the litters were genotyped, their ovaries excised at ~4 weeks of age, and GV oocytes collected. Since GLYT1 is maximally activated in MII eggs [18], the oocytes were cultured in MEMα medium overnight before the rate of glycine transport was measured in each individual egg. Twelve female offspring in total were obtained from the three pairs for these experiments, seven of which were Gdf9-iCre:Glyt1^fl/fl^ and five of which were Glyt1^fl/fl^ (Figure 1A). All oocytes from Gdf9-iCre:Glyt1^fl/fl^ females exhibited low rates of glycine transport that were approximately the level expected for non-specific transport, while all oocytes from Glyt1^fl/fl^ had high rates of glycine transport (Figure 1B). Thus, oocytes with *Slc6a9* disrupted did not transport glycine, consistent with essentially all glycine transport in oocytes being mediated by GLYT1. This also independently confirms that all GV oocytes in females with the Gdf9-iCre:Glyt1^fl/fl^ genotype have the expected *Slc6a9*^−/−^ phenotype of no GLYT1 activity. Because of the limited number of females that could be produced and the ability to directly demonstrate the functional knockout of glycine transport in single oocytes, we did not also attempt to confirm the knockout by measuring *Slc6a9* transcripts or protein, which require large pools of oocytes.

### 3.2. Fertility of Gdf9-iCre:Glyt1^fl/fl^ Females

To determine if Gdf9-iCre:Glyt1^fl/fl^ females, whose eggs lacked GLYT1 activity, were fertile, those females or control Glyt1^fl/fl^ genotype females (N = 3 each) were paired with wild type BDF1 males until they had three litters. The Glyt1^fl/fl^ females had three litters in 68, 78, and 78 days, while the Gdf9-iCre:Glyt1^fl/fl^ had three litters in 96, 64, and 89 days (mean ± s.e.m. = 74.7 ± 3.3 and 83.0 ± 9.7 days, respectively). The mean times to produce three litters were not significantly different (*p* = 0.46, *t*-test). The mean number of pups per litter were 7.56 ± 0.63 for Glyt1^fl/fl^ and 7.67 ± 0.97 for Gdf9-iCre:Glyt1^fl/fl^, which was not significantly different (*p* = 0.92, *t*-test). This indicated that oocytes that lacked GLYT1 activity had normal developmental potential in vivo.

### 3.3. Attempts to Delete GLYT1 Activity from Cumulus Cells

Because the deletion of GLYT1 activity from oocytes did not affect female fertility, we asked whether oocytes could be accumulating glycine through other routes. The cumulus cells surrounding oocytes also transport glycine mainly via GLYT1 and can supply glycine to the enclosed oocyte via the gap junctions that couple the oocyte to the cumulus [35]. Glycine transport into COCs becomes active when ovulation is triggered or the COC is removed from the follicle, similar to denuded oocytes [18,19]. Therefore, it was possible that oocytes that lacked their own GLYT1 activity could be supplied with glycine in vivo via cumulus cells.

**Figure 2:**
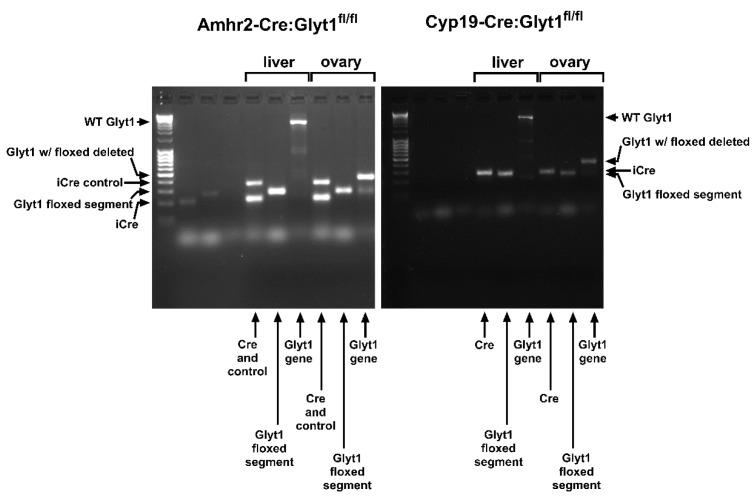
Assessing the deletion of the floxed segment of *Slc6a9* in Amhr2-Cre:Glyt1^fl/fl^ or Cyp19-Cre:Glyt1^fl/fl^ ovaries. Whole ovaries from Amhr2-Cre:Glyt1^fl/fl^ or Cyp19-Cre:Glyt1^fl/fl^ female mice (as indicated above gels) were assessed by PCR. MassRuler DNA ladders are shown at left. Liver from the same females was used as a control. Amhr2-Cre:Glyt1^fl/fl^: For both liver and ovary, the presence of the Amhr2-Cre transgene was confirmed by the iCre band at 200 bp obtained with Jackson Laboratory Cre primers (see Materials and Methods) multiplexed with their internal control primers (oIMR7338 and oIMR7339; 324 bp; labeled “Cre and control”). As expected, the Amhr2-Cre transgene was present in both liver and ovary (“iCre” arrow at left). The floxed segment of *Slc6a9* was confirmed using the 17854 and 17672 primers that yield a 258 bp product for Glyt1^fl/fl^ (labeled “Glyt1 floxed segment” and corresponding arrow at left). Deletion of the floxed segment of the *Slc6a9* gene was assessed using the o250 and o228 primer pair (labeled “Glyt1 gene”). In liver, only the ~6,600 bp product corresponding to the full-length gene was amplified (“Glyt1 WT” arrow at left). In contrast, only a ~450 bp band corresponding to the *Slc6a9* gene with the floxed segment deleted (“Glyt1 w/ floxed deleted” arrow at left) was amplified from ovary. Full-length *Slc6a9* would also be present in ovary but cannot be detected here due to the overwhelming preferential amplification of the ~450 bp product. Together, this confirms that the Amhr2-Cre transgene is present, that the tissues are Glyt1^fl/fl^, and that no detectable *Slc6a9* deletion occurred in liver but was present in ovary. The extent of the deletion in ovary is unknown, however. Cyp19-Cre:Glyt1^fl/fl^: Lanes are labeled as for Amhr2-Cre:Glyt1^fl/fl^. The Cyp19-Cre transgene was confirmed in both liver and ovary with the Cp19CreF and Cp19CreR primer pair that yields a 290 bp product. No control gene was used. Primers for Glyt1^fl/fl^ and detecting deletion of the floxed segment of *Slc6a9* are as for Amhr2-Cre. The presence of the Cyp19-Cre transgene was confirmed (labeled “Cre” and “iCre” arrow at right). As for Amhr2-Cre, both tissues were Glyt1^fl/fl^ (labeled “Glyt1 floxed segment” and corresponding arrow at right). No detectable *Slc6a9* deletion occurred in liver but was present in ovary (labeled “Glyt1 gene” with “WT Glyt1” and Glyt1 w/ floxed deleted” arrows at right). As for Amhr2-Cre, the extent of deletion in ovary is not known (see above). In each panel, the first three lanes are ear notch samples that failed to yield a sufficient signal.

**Figure 3:**
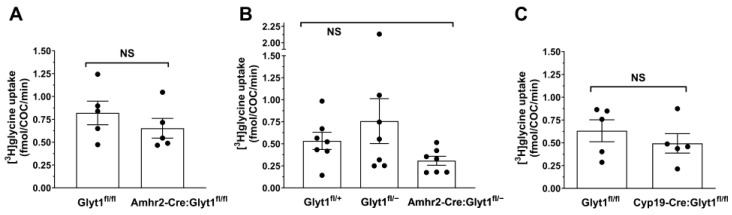
Glycine transport into cumulus-oocyte complexes. (**A**) Glycine transport was measured in COCs from females of Glyt1^fl/fl^ or Amhr2-Cre: Glyt1^fl/fl^ genotypes. There was no significant difference in the rates of glycine transport between genotypes (*p* = 0.35 by unpaired *t*-test). Each point represents the uptake from 4–10 pooled COCs from one female (expressed as rate per COC; numbers vary since all intact COCs from a female were used). The bars indicate the mean ± s.e.m. for 5 females in each group. (**B**) Glycine transport was measured in COCs from females of Glyt1^fl/fl^ or Amhr2-Cre: Glyt1^fl/fl^ genotypes as in (**A**) except that they were on an *Slc6a9*^+/˗^ background as an attempt to obtain a more complete knockout in cumulus cells. There was no significant difference between the rates of glycine transport between genotypes (*p* = 0.17 by one-way ANOVA comparing all three groups). Each point represents the uptake from 4–23 pooled COCs from one female as in (**A**). The bars indicate the mean ± s.e.m. for 7 females in each group. (**C**) Glycine transport was measured in COCs from females of Glyt1^fl/fl^ or Cyp19-Cre: Glyt1^fl/fl^ genotypes as in (**A**,**B**). There was no significant difference between the rates of glycine transport between genotypes (*p* = 0.42 by unpaired *t*-test). Each point represents the uptake from 2–13 pooled COCs from one female as in (**A**,**B**). The bars indicate the mean ± s.e.m. for 5 females in each group.

We therefore attempted to create a mouse model in which glycine transport into granulosa cells was deleted by interbreeding Amhr2-Cre (which is expressed in cumulus cells [29,30]) and Glyt1^fl/fl^ lines to produce Amhr2-Cre:Glyt1^fl/fl^ mice. Detection of the Glyt1^fl/fl^ deletion of a segment of *Slc6a9* was confirmed in whole ovaries (Figure 2). We then isolated COCs from Amhr2-Cre:Glyt1^fl/fl^ females and Glyt1^fl/fl^ controls and compared the rates of glycine transport. Despite the presence in ovaries of the *Slc6a9* gene in which the region flanked by LoxP sequences had been deleted, the rates of glycine transport into COCs from these females was not significantly lower than transport into COCs from Glyt1^fl/fl^ controls (Figure 3A).

We reasoned that this might be due to the need to delete the LoxP-flanked segments of both copies of the *Slc6a9* gene from the majority of cumulus granulosa cells surrounding each oocyte to eliminate glycine transport into the COC. To increase the chance of a complete knockout of both copies of the gene, we produced Amhr2-Cre:Glyt1^fl/−^ mice that had one copy of the *Slc6a9* gene disrupted and the other floxed. These were mated with Amhr2-Cre:Glyt1^fl/fl^ mice to produce Amhr2-Cre:Glyt1^fl/−^ females and also Glyt1^fl/-^ and Glyt1^fl/+^ control females. COCs from *Slc6a9* heterozygous (Glyt1^fl/−^) and wild type (Glyt1^fl/fl^) females exhibited similar rates of glycine transport into their COCs (Figure 3B). There was some trend towards lower glycine transport into COCs from Amhr2-Cre:Glyt1^fl/−^ females, which was not significant, and there was still considerable glycine transport into these COCs (Figure 3B). This indicated that expression of Amhr2-Cre was not sufficient to eliminate glycine transport even from Glyt1^fl/−^ cumulus cells.

We next attempted to eliminate glycine transport into cumulus cells using Cyp19-Cre, which is also expressed in cumulus cells [31,32]. Cyp19-Cre:Glyt1^fl/fl^ females were produced and glycine transport into their COCs was measured. Detection of the Glyt1^fl/fl^ deletion was confirmed in whole ovaries (Figure 2). However, similar to the results with Amhr2-Cre, glycine transport into COCs from Cyp19-Cre:Glyt1^fl/fl^ females was not significantly decreased compared to Glyt1^fl/fl^ controls (Figure 3C). Thus, it did not prove possible to produce a substantial decrease in glycine transport into cumulus cells, in contrast to oocytes.

### 3.4. Transport of Glycine from Cumulus Cells into Oocytes

**Figure 4:**
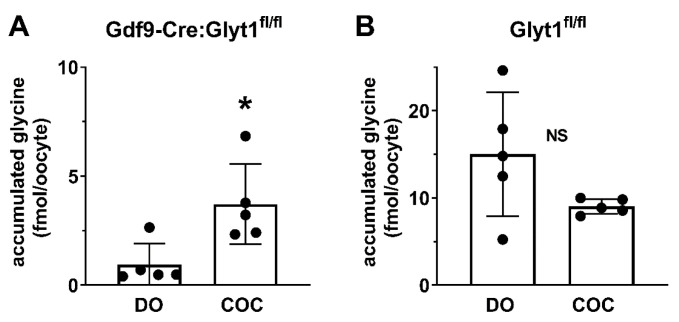
Transfer of glycine to oocytes with cumulus-oocyte complexes. (**A**) COCs and denuded oocytes from Gdf9-Cre:Glyt1^fl/fl^ females were incubated with [^3^H]glycine for 4 h, after which the oocytes were removed from the COC and the amount of [^3^H]glycine in oocytes was determined. Denuded oocytes from Gdf9-Cre:Glyt1^fl/fl^ females accumulated low levels of glycine, while oocytes that had been within COCs accumulated significantly higher amounts of glycine (* *p* = 0.017, unpaired *t*-test), indicating that glycine was transferred into the enclosed oocyte from the cumulus. (**B**) Glycine accumulation in oocytes was determined as in (**A**) except the oocytes and COCs were from Glyt1^fl/fl^ females. As expected, denuded oocytes accumulated glycine. The amount of glycine accumulated was not significantly different between oocytes that had been enclosed in COCs and denuded oocytes (*p* = 0.10, unpaired *t*-test). In both panels, each point represents a pool of 6–18 oocytes with the bars indicating the mean ± s.e.m.

As described above, we were not able to knock out glycine transport into cumulus cells, which would have allowed the production of a double knockout of glycine transport in oocytes and cumulus. However, we could nonetheless test the hypothesis that wild type cumulus cells can supply glycine to enclosed oocytes that themselves lack glycine transport. We collected COCs from Gdf9-iCre:Glyt1^fl/fl^ females, which contain oocytes that cannot transport glycine and from Glyt1^fl/fl^ females with oocytes that can transport glycine (see Figure 1). COCs and denuded oocytes were incubated for 4 h with [^3^H]glycine. The relevant comparison is between the amount of glycine that was accumulated in the conditional knockout oocytes from Gdf9-iCre:Glyt1^fl/fl^ females that were within cumulus cells vs. those that were cultured as denuded oocytes. As predicted from the results above, denuded oocytes from Gdf9-iCre:Glyt1^fl/fl^ females accumulated glycine to a very low level (not significantly different than zero, one-factor *t*-test, *p* = 0.093). However, those that were connected to cumulus cells during culture accumulated significantly more glycine (Figure 4A; *p* = 0.017 by unpaired *t*-test). Thus, oocytes which lack GLYT1 activity can accumulate glycine when they are within COCs. In contrast, there was no difference in glycine accumulation when COCs and oocytes from wild type Glyt1^fl/fl^ females were used (Figure 4B; *p* = 0.10 by unpaired *t*-test).

### 3.5. Effect of Increased Osmolarity on Embryos from Oocytes of Gdf9-iCre:Glyt1^fl/fl^ Females

**Figure 5:**
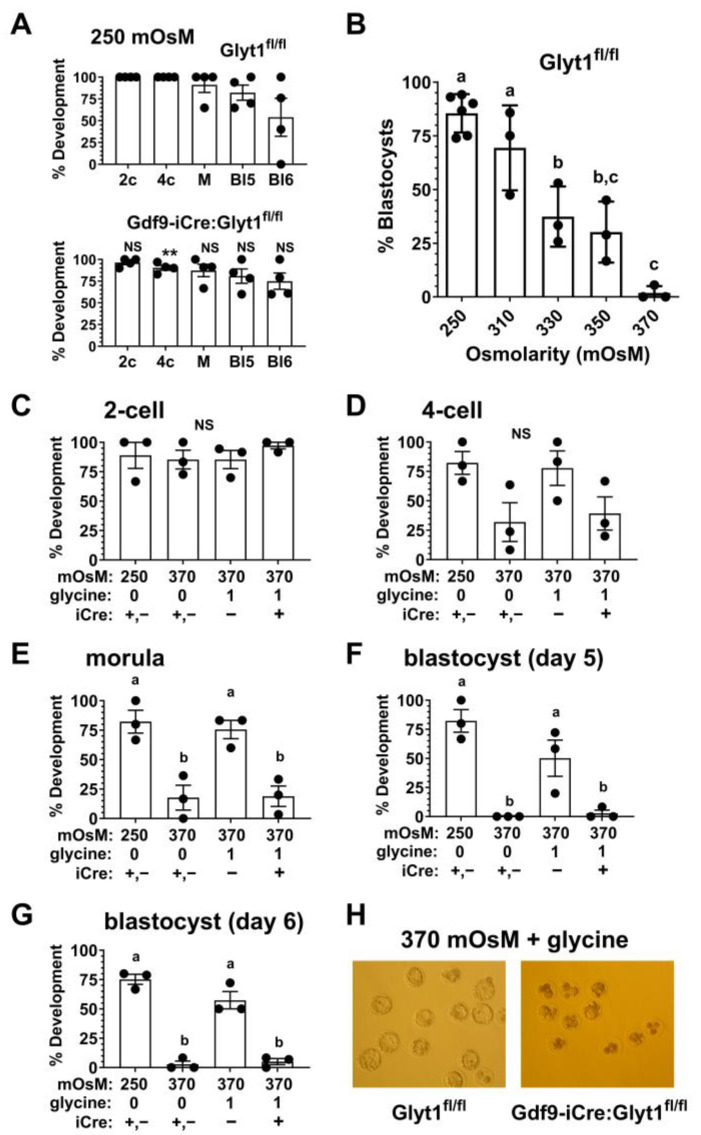
Development of 1-cell embryos from Gdf9-Cre:Glyt1^fl/fl^ females at increased osmolarity. (**A**) One-cell embryos from either Glyt1^fl/fl^ or Gdf9-Cre:Glyt1^fl/fl^ females developed similarly in normal mKSOM medium whose osmolarity is 250 mOsM. The stages of development are indicated as 2-cell (2c), 4-cell (4c), morula (M), blastocyst on day 5 of culture (Bl5), and blastocyst on day 6 (Bl6). The difference between development at each stage was tested between genotypes. The difference at the 4-cell stage reached significance (** *p* = 0.015, unpaired *t*-test), while all others were not significantly different. This indicated that the genotype did not affect normal preimplantation development. In all panels, each point represents the development of a group of ~10 embryos and the bars indicate the mean ± s.e.m. (**B**) The dependence of preimplantation development from 1-cell embryos to blastocysts was assessed as a function of osmolarity. Development decreased with osmolarity with a significant decrease observed by 330 mOsM and almost no development at 370 mOsM (*p* < 0.0001 by one-way ANOVA with Tukey’s multiple comparisons test; bars that do not share the same letter are significantly different, *p* < 0.05). There were three independent repeats at each osmolarity except for 250 mOsM (N = 5). (**C**) Development from the 1-cell to 2-cell stages was assessed at 250 mOsM and at 370 mOsM in the absence of glycine (0) or with 1 mM glycine (1) in the medium. The genotypes of the females from which the embryos were derived were either pooled Glyt1^fl/fl^ and Gdf9-iCre:Glyt1^fl/fl^ (iCre +,-), Gdf9-iCre:Glyt1^fl/fl^ (+), or Glyt1^fl/fl^ (-). Development was not significantly different between groups (*p* = 0.69 by one-way ANOVA). (**D**) Development from the 1-cell to 4-cell stages was assessed. Groups are as in (**C**). The differences in development to the 4-cell stage did not reach significance (*p* = 0.07 by one-way ANOVA). Development from the 1-cell to the morula stage (**E**) and blastocyst stages on day 5 (**F**) and day 6 (**G**) was assessed. Groups are as in (**C**). Development to each stage was significantly decreased at 370 mOsM (*p* < 0.005 by one-way ANOVA with Tukey’s multiple comparisons test). Glycine in the culture medium completely rescued development in embryos from Glyt1^fl/fl^ females (−) to each stage. Development was not significantly different than at 250 mOsM (*p* = 0.95 for morula, 0.15 for blastocysts on day 5, and *p* = 0.10 on day 6). However, glycine did not rescue development of embryos derived from Gdf9-iCre:Glyt1^fl/fl^ females (+), which were significantly different from those at 250 mOsM (*p* = 0.006 for morula, *p* = 0.001 for blastocysts on day 5, *p* < 0.0001 day 6) and from Glyt1^fl/fl^ at 370 mOsM in the presence of glycine (*p* = 0.01 for morula, *p* = 0.03 for blastocysts on day 5, *p* = 0.0002 day 6). For E-G, bars that do not share the same letter are significantly different, *p* < 0.05 or as specified above. (**H**) Examples of blastocysts developed from 1-cell embryos derived from Glyt1^fl/fl^ or Gdf9-iCre:Glyt1^fl/fl^ females (as indicated at bottom) cultured at 370 mOsM with 1 mM glycine. Glycine rescued development to the blastocyst stage of Glyt1^fl/fl^ embryos with GLYT1 activity. However, Gdf9-iCre:Glyt1^fl/fl^ embryos lacking GLYT1 activity degenerated under the same conditions. For scale, expanded blastocysts are ~100 µm in diameter.

It appears that oocytes lacking functional GLYT1 can likely accumulate some glycine in vivo via transport by coupled cumulus cells. However, in vitro, oocytes lacking GLYT1 activity would be predicted to be more sensitive to increased osmolarity. We first confirmed that the loss of functional GLYT1 had no effect on development in vitro at the normal osmolarity of mKSOM medium, 250 mOsM. There was no effect on development to the 2-cell, morula, or blastocyst stages at 250 mOsM (Figure 5A). There was, however, a significant decrease in development to the 4-cell stage, although this was a small effect of uncertain biological significance.

Previously, we had shown that strains of mice differed in their sensitivity to hypertonic stress [2], and the response of embryos from C57Bl/6 females to increased osmolarity was not known. We therefore tested the ability of 1-cell embryos from Glyt1^fl/fl^ females on the C57Bl/6 background to develop to blastocysts in vitro in media with increased osmolarities. Embryos developed to the blastocyst stage similarly at the normal osmolarity of mKSOM (250 mOsM) and at 310 mOsM. However, development decreased significantly between 310 and 330 mOsM and very few embryos developed to blastocysts at 370 mOsM (Figure 5B). To most effectively reveal any ability of glycine to rescue development, we carried out subsequent experiments at 370 mOsM.

We then assessed the development of 1-cell embryos to the 2-cell stage (day 2 of culture), 4-cell or greater (day 3), morula (day 4), and to the blastocyst stage on days 5 or 6 (Figure 5C–G). At 250 mOsM, embryos developed to the blastocyst stage in the absence of added glycine, while little or no development to the 4-cell stage and beyond occurred in 370 mOsM medium without glycine. Due to the need to conserve embryos, we pooled 1-cell embryos derived from Glyt1^fl/fl^ and Gdf9-iCre:Glyt1^fl/fl^ females (indicated by iCre +,−; Figure 5C–G), since these developed similarly in culture in the absence of added glycine and the presence of the iCre transgene had no effect by itself (Figure 5A). This confirmed the expected developmental arrest after the 2-cell stage at high osmolarity. The presence of glycine in the culture medium rescued development to the blastocyst stage of 1-cell embryos derived from oocytes of Glyt1^fl/fl^ females (iCre −), which had active GLYT1 (Figure 5C–G). However, even in the presence of glycine, 1-cell embryos derived from oocytes of Gdf9-iCre:Glyt1^fl/fl^ females (iCre +), which lack GLYT1 activity, failed to develop beyond the 4-cell stage, and very few reached the blastocyst stage (Figure 5D–G). These embryos instead degenerated in culture (Figure 5H). Thus, GLYT1 is required in oocytes for subsequent embryo development to be rescued from hypertonic stress by glycine.

## 4. Discussion

Glycine is the major organic osmolyte in preimplantation mouse embryos, where it plays a key role in maintaining cell volume [7,15,23,36]. It likely has a similar role in the embryos of other mammals including humans [22]. In the absence of organic osmolytes supplied from the external environment, relatively minor increases in osmolarity can result in developmental arrest in early embryogenesis [1,2,4,14,16]. It had been established that glycine is transported into mouse embryos during the 1-cell to 4-cell stages by a transporter that had the characteristics of the classic System Gly that is mediated by the GLYT1 transporter encoded by *Slc6a9*. The evidence for this included competitive inhibition by sarcosine, dependence on both Na^+^ and Cl^˗^, and inhibition by a small molecule inhibitor, ORG23798, that is selective for GLYT1 [15,16,23]. However, it had not been shown that a functional *Slc6a9* gene is required for glycine transport into early preimplantation embryos. Here, we were able to create an oocyte-specific knockout of *Slc6a9* by crossing mice in which a segment of the gene had been flanked by LoxP sites [25,26] with transgenic mice expressing iCRE driven by the *Gdf9* promoter [27]. Offspring of Gdf9-iCre:Glyt1^fl/fl^ females mated to wild type males were all heterozygous for *Slc6a9*, indicating that all oocytes that gave rise to these offspring were *Slc6a9*^−/−^. Furthermore, when matured, such oocytes all failed to transport glycine, confirming that *Slc6a9* in necessary for the robust glycine transport found in eggs and persisting in early preimplantation embryos.

Unexpectedly, female Gdf9-Cre:Glyt1^fl/fl^ females were fertile, producing litters whose sizes and frequency were indistinguishable from those of Glyt1^fl/fl^ females. There are several possible explanations for this result. The first is that glycine is not required by oocytes and early preimplantation embryos in vivo. There is considerable evidence that glycine is used by mouse embryos as an organic osmolyte and rescues development from the developmental block that occurs when osmolarity is increased in vitro [2,7,14,15,23,36]. However, it had not been shown that glycine is similarly required for preimplantation development in vivo. It is possible that it is used by embryos only under conditions of osmotic stress (e.g., dehydration), but this would not explain why embryos contain such high levels of endogenous glycine [18] when mice were housed under normal laboratory conditions with ad libitum access to water.

A second possibility is that glycine is supplied to embryos through another route that compensates for the loss of GLYT1 activity in the embryos themselves. We showed here that glycine taken up by cumulus cells can supply glycine to the enclosed oocyte within a COC, which supports the hypothesis that cumulus-mediated glycine transport can compensate at least partly for the loss of GLYT1 in the egg and early preimplantation embryo. The large majority of glycine transport by the cumulus is also via GLYT1 [35]. However, attempts to eliminate GLYT1 activity in cumulus cells by disrupting the *Slc6a9* gene in granulosa cells using Cre expression driven by the *Amhr2* promoter in either homozygous Glyt1^fl/fl^ females or heterozygous Glyt1^fl/−^ females lacking one copy of the *Slc6a9* gene were unsuccessful, as were attempts using Cre expression driven by the *Cyp19a1* promoter. Therefore, it could not be determined whether eliminating both routes of glycine accumulation via GLYT1 would affect female fertility. However, the accumulation of glycine in *Slc6a9*^−/−^ oocytes within COCs supports the hypothesis that GLYT1 in cumulus cells compensates for its loss in eggs and early embryos and that it may contribute to maintaining female fertility.

A third possibility is that organic osmolytes other than glycine contribute to volume regulation of eggs and early embryos. In addition to glycine, betaine (N,N,N-trimethylglycine) is a major osmolyte in mouse eggs and preimplantation embryos. It is synthesized in oocytes during meiotic maturation by the enzyme choline dehydrogenase (CHDH) where it is accumulated to very high levels in the mature egg [37]. Its intracellular concentration is then regulated in 1-cell and 2-cell embryos by the SIT1 (*Slc6a20a*) transporter [38,39]. Thus, increased betaine synthesis or transport could compensate for decreased glycine in embryos. Whether knocking out the betaine pathways in conjunction with knocking out GLYT1 activity affects female fertility remains to be determined, however.

Another consideration is that different strains of mice have very different sensitivities to increased osmolarity. Mice of different strains exhibit widely varying susceptibility to the classic “2-cell block” to development, ranging from those that suffer a near-total block in traditional embryo culture media to classic “non-blocking” strains that developed to blastocysts in vitro at high rates [40,41]. We had previously shown that the block that occurred when osmolarity was increased was identical to the 2-cell block, with some strains becoming blocked at near-oviductal osmolarities, while others only became blocked at much higher osmolarities [2,4]. The C57Bl6 mice used in the current investigations become blocked at intermediate osmolarities beginning around 330 mOsM, between sensitive strains that become blocked at <310 mOsM and resistant strains that only become blocked at >350 mOsM. It is possible, therefore, that the fertility of more sensitive strains might be affected by lack of GLYT1 activity in oocytes. This remains to be investigated.

A requirement for GLYT1 activity in preimplantation embryos for glycine to mitigate against the deleterious effects of increased osmolarity was confirmed. As was previously shown, 1-cell stage embryos would not develop at increased osmolarity, but development could be rescued with glycine added to the medium [2,23]. The ability of glycine to rescue development was lost, however, in embryos in which *Slc6a9* was disrupted, conclusively demonstrating the requirement for GLYT1. Although we used 370 mOsM medium here to obtain a maximum difference between development with and without glycine, a significant decrease in development was evident between 310 and 330 mOsM. This is nearer to the in vivo osmolarity of oviductal fluid, which has been measured to be ~300 mOsM [42,43,44], although calculations have indicated it may reach as high as 350 mOsM during early preimplantation embryo development [3]. This is supportive of a possible role for glycine in vivo, since relatively small increases in oviductal fluid osmolarity would have an impact on embryo development even at the lower values proposed for oviductal fluid. A role for glycine in vivo is also implied by the large intracellular concentration of glycine in the egg through 2-cell embryo stages [18,21], which can be maintained against a steep outward gradient only at considerable and continuous metabolic cost.

Ample evidence suggests that organic osmolytes such as glycine are beneficial to early preimplantation embryos in vitro [1]. This likely includes human embryos, since they transport glycine via GLYT1 similarly to mouse embryos [22]. Current embryo culture media in use in the clinic include glycine and other amino acids and, therefore, support cell volume regulation that requires organic osmolytes. The results presented here indicate the need for glycine in media used for in vitro oocyte maturation as well, to support the accumulation of glycine in the mature egg.

In summary, we have shown conclusively that a functional *Slc6a9* gene is required for GLYT1 activity in mouse oocytes. Additionally, it is required for the rescue by glycine of development at increased osmolarities in vitro. At least in the C57Bl6 strain, GLYT1 in oocytes and preimplantation embryos is not required for female fertility. We propose that this is likely due to redundant mechanisms that include glycine transport into oocytes via cumulus cells and compensation by other organic osmolytes such as betaine. Future studies will be needed to develop a method of also knocking out glycine transport in cumulus cells, to determine whether this would decrease fertility. Also, it remains to be determined whether preventing the use of other organic osmolytes such as betaine in oocytes in conjunction with knocking out GLYT1 activity results in impaired fertility.

## Data Availability

Data generated and analyzed for this study are included in this article. Unprocessed original data are available upon reasonable request to the corresponding author (J.M.B.).
